# IFI16 Protein Mediates the Anti-inflammatory Actions of the Type-I Interferons through Suppression of Activation of Caspase-1 by Inflammasomes

**DOI:** 10.1371/journal.pone.0027040

**Published:** 2011-10-28

**Authors:** Sudhakar Veeranki, Xin Duan, Ravichandran Panchanathan, Hongzhu Liu, Divaker Choubey

**Affiliations:** 1 Department of Environmental Health, University of Cincinnati, Cincinnati, Ohio, United States of America; 2 Cincinnati VA Medical Center, Cincinnati, Ohio, United States of America; Universidade de Sao Paulo, Brazil

## Abstract

**Background:**

Type-I interferons (IFNs) are used to treat certain inflammatory diseases. Moreover, activation of type-I IFN-signaling in immune cells inhibits the production of proinflammatory cytokines and activation of inflammasomes. However, the molecular mechanisms remain largely unknown. Upon sensing cytosolic double-stranded DNA, the AIM2 protein forms the AIM2-ASC inflammasome, resulting in activation of caspase-1. Given that the IFI16 and AIM2 proteins are IFN-inducible and can heterodimerize with each other, we investigated the regulation of IFI16, AIM2, and inflammasome proteins by type-I and type-II IFNs and explored whether the IFI16 protein could negatively regulate the activation of the AIM2 (or other) inflammasome.

**Methodology/ Principal Findings:**

We found that basal levels of the IFI16 and AIM2 proteins were relatively low in peripheral blood monocytes (CD14^+^) and in the THP-1 monocytic cell line. However, treatment of THP-1 cells with type-I (IFN-α or β) or type-II (IFN-γ) IFN induced the expression levels of IFI16, AIM2, ASC and CASP1 proteins. The induced levels of IFI16 and AIM2 proteins were detected primarily in the cytoplasm. Accordingly, relatively more IFI16 protein bound with the AIM2 protein in the cytoplasmic fraction. Notably, increased expression of IFI16 protein in transfected HEK-293 cells inhibited activation of caspase-1 by the AIM2-ASC inflammasome. Moreover, the constitutive knockdown of the *IFI16* expression in THP-1 cells increased the basal and induced [induced by poly(dA:dT) or alum] activation of the caspase-1 by the AIM2 and NLRP3 inflammasomes.

**Conclusions/Significance:**

Our observations revealed that the type-I and type-II IFNs induce the expression of IFI16, AIM2, and inflammasome proteins to various extents in THP-1 cells and the expression of IFI16 protein in THP-1 cells suppresses the activation of caspase-1 by the AIM2 and NLRP3 inflammasomes. Thus, our observations identify the IFI16 protein as a mediator of the anti-inflammatory actions of the type-I IFNs.

## Introduction

The interferons (IFNs) are a family of cytokines [1], [2]. The family includes type-I (IFN-α and β), type-II (IFN-γ), and type III IFNs [1], [3]. IFNs exert multiple biological effects on cells through binding to cell surface receptor and activating the IFN-signaling [1]. The binding of the type I IFNs (α and β) to the cell surface receptor results in activating phosphorylation of signal transducer and activator of transcription 1 (STAT1) protein in the cytoplasm, which then translocates to the nucleus and activates the transcription of the IFN-inducible genes, such as the *IFI16* and *AIM2* [4]. The proteins encoded by the IFN-inducible genes mediate various biological and immunomodulatory activities of the IFNs [1], [5].

Most cell types produce low constitutive levels of type I IFNs [2]. However, their expression is induced as a part of an innate immune response that is initiated after infections [1], [2]. Type I IFNs are used to treat certain autoimmune and inflammatory diseases, such as multiple sclerosis (MS) [6], familiar Mediterranean fever (FMF) [7], and Behcet's syndrome [8]. Notably, increased serum levels of type I IFN (IFN-α) in individuals are associated with increased risk to develop systemic lupus erythematosus (SLE) [9], [10], an autoimmune disease with systemic inflammation [11]. The above observations are consistent with an anti-inflammatory as well as an inflammatory role for the type I IFNs. However, the molecular mechanisms remain largely unknown.

The IFN-inducible p200-family proteins are encoded by the murine (for example, *Ifi202a*, *Ifi202b*, and *Aim2*) and human (for example, *IFI16* and *AIM2*) genes [4], [12]. These proteins share either one or two partially conserved repeats of 200-amino acid residues (HIN-200-aa domain) towards the C-terminus. The domain contains two consecutive oligonucleotide/ oligosaccharide-binding folds (OB-folds) [13], which allow binding to double-stranded DNA (dsDNA) in sequence independent manner [14]. Surprisingly, only the Aim2 protein in the family is conserved (55% amino acid identities) between mice and humans [15]. Most p200-family proteins (except the p202a and b proteins) also contain a PYRIN domain (PYD) in the N-terminus [15]. The PYD in the AIM2 protein allows heterodimerization with other p200-family proteins, including the p202 [16] and IFI16 [17] proteins. Given that most of the p200-family proteins contain both PYD and HIN200 domains, these proteins are also referred as the PYHIN proteins [18].

Several recently identified proteins sense cytosolic double-stranded DNA (dsDNA). These proteins include DAI [19], RNA polymerase III [20], and LRRFIP1 [21]. Additionally, the AIM2 [22]-[24] and IFI16 [25] PYHIN proteins can also sense cytosolic DNA. Moreover, the AIM2 protein is required for innate immune recognition of *Francisella tularensis* [26]. Upon sensing cytosolic dsDNA, the AIM2 protein undergoes a conformational change and recruits apoptosis speck like protein containing a CARD (ASC) domain through its PYD. The ASC protein then interacts with caspase-1 through its CARD domain. The resulting protein complex, which is termed inflammasome [27], serves as a molecular platform that mediates the autoactivation of caspase-1, resulting in a ∼20 kDa (p20) protein fragment. When activated, the caspase-1 cleaves the pro-forms of the inflammatory cytokines, such as IL-1β and IL-18, to active forms [27]. Interestingly, type I interferon signaling is required for the activation of the AIM2 inflammasome during *Francisella tularensis* infection [24]. Excessive secretion of IL-1β and IL-18 cytokines is linked to an increasing number of human inflammatory diseases [28]. For example, an inflammasome is constitutively activated with cleavage of caspase-1 in human melanoma cells [29]. In contrast to the constitutive activation of an inflammasome, the lack of activation is also predicted to result in defective innate immune responses, increased constitutive production of the IFN-β, and the development of autoimmunity [30], [31].

Upon sensing cytosolic dsDNA, the IFI16 protein is reported to recruit the stimulator of interferon genes (STING) protein to stimulate the expression of IFN-β through the activation of the transcriptional activity of IRF3 and NF-κB in macrophage cell line THP-1 [25]. In contrast, upon infection of endothelial cells with Kaposi sarcoma-associated herpesvirus (KSHV), the IFI16 protein is reported to sense the viral DNA in the nucleus to induce the activation of caspase-1 and inflammasome [32]. Notably, increased levels of IFI16 protein in human normal diploid fibroblasts (HDFs) [33] and prostate epithelial cells [34] are associated with the onset of cellular senescence. Interestingly, reduced levels of the IFI16 protein in senescent (versus young or old) HDFs are associated with senescence-associated secretory phenotype (SASP) [35]. The secretory phenotype in the senescent cells is associated with the activation of NF-κB activity and the expression of target genes, which encode for the proinflammatory cytokines [36].

A recent study indicated that activation of type I IFN-signaling in bone marrow-derived macrophages (BMDMs) can inhibit the production of interleukin-1 (IL-1) by inflammasomes, such as the NLRP1 and NLRP3, through the STAT1-dependent mechanism [37]. Additionally, the study showed that the activation of type I IFN-signaling in BMDMs reduced the constitutive levels of the pro-IL-1β, thus, explaining the effectiveness of the type I IFN (the IFN-β) in the treatment of the inflammatory diseases [37]. However, the IFN-inducible protein(s) that mediates the anti-inflammatory functions of the type I IFN remain to be identified.

Given that the expression of both AIM2 and IFI16 innate immune sensors for the cytosolic DNA is induced by type I IFNs in certain cell types and the activation of type I IFN-signaling can inhibit the production of IL-1β [37], we explored the potential role of the IFI16 protein in the regulation of the AIM2 inflammasome. Here we report that the type I and II IFNs up-regulate the expression of IFI16 and AIM2 proteins to different extents and that the IFI16 protein suppresses the activation of caspase-1 by the AIM2 and NLRP3 inflammasomes.

## Materials and Methods

### Cells and Treatments

Frozen human peripheral blood mononuclear cells (PBMCs), purified CD14+ monocytes and CD19+ B cells were purchased from ReachBio (Seattle, WA). THP-1 cells were purchased from the American Type Culture Collection (ATCC; Manassas, VA). Cultures of THP-1 cells were maintained (CO_2_, 5.5%) in RPMI-1640 culture medium (cat # 30-2001, purchased from ATCC) with high glucose, which was supplemented with 10% fetal bovine serum and antibiotics (Invitrogen, Carlsbad, CA). Differentiation of THP-1 cells was induced as described previously [24]. Briefly, THP-1 cells were incubated with 1 mM PMA for 3 h or 20 nM for overnight and then the cells were washed with PBS and were allowed to adhere to the surface of the tissue culture plates. Human embryonic kidney cells (HEK-293T) were purchased from ATCC and maintained in DMEM with high glucose (Invitrogen). The medium was supplemented with 10% fetal bovine serum (Invitrogen).

PMA differentiated THP-1 cells were primed with LPS (100 ng/ml, Sigma) for 3–4 h, followed by stimulation of the NLRP3 inflammasome with alum (Sigma; 300 µg/ml for 150 min) as described previously [37].

### Transfections and Expression Vectors

Transient transfections in HEK-293T cells were performed using Lipofectamine 2000 (Invitrogen #11668-027) as suggested by the supplier. The transfected cells were harvested after 40–45 h of transfections. The following expression plasmids, which allowed the expression of the FLAG-tagged proteins (the tag at the C-terminus of the proteins), were purchased from OriGene (Rockville, MD): hAIM2 (NM_004833), hASC (NM_013258), and Caspase1 (NM_033293). The pCDNA3-IFI16B plasmid has been described previously [34].

### Nucleofections

THP-1 cells (∼2×10^6^ cells/ nucleofection) were nucleofected as described previously (37). In brief, cells were nucleofected with 2 µg of synthetic DNA [poly(dA:dT); from InvivoGen, San Diego, CA] using the Nucleofector-II device (Amaxa Biosystems, Natthermannalle 1, Germany). We used nucleofection kit-V and program U-001. These nucleofection conditions resulted in ∼60% cell survival. After nucleofections, cells were harvested at the indicated time for immunoblotting.

### Knockdown of IFI16 Expression

THP-1 cells were either infected with control lentiviral particles (sc-108080; from Santa Cruz Biotech, Santa Cruz,, CA) or the lentiviral particles expressing shIFI16 RNA (sc-35633-V, Santa Cruz Biotech) in a six well plate as suggested by the supplier. A day after infections, cells were selected with puromycin (0.5–1 µg/ml) for about a week. The selected cells were pooled and maintained in puromycin (0.5 µg/ml) for another week. At the end of puromycin selection, cells were serially diluted to isolate clonal cell populations. Several independent cell populations were screened for the knockdown of IFI16 expression by quantitative real-time PCR as well as immunoblotting. We were able to identify at least three clonal cell populations in which we achieved ∼80–90% knockdown of IFI16 expression. For experiments, cells were cultured without puromycin for at least three days.

### Immunoblotting

Total cell lysates were prepared and analyzed by immunoblotting as described previously [38]. Endogenous AIM2 protein was detected by rabbit polyclonal antibodies that were raised against the KLH conjugated hAIM2 C-terminal peptide (GVHSTIKVIKAKKKT). This antibody preparation detected a protein band ∼48 kDa, which was induced by treatment of cells with interferon [35]. Antibodies specific for IFI16 (sc-8023) and ASC (sc-22514) proteins were purchased from Santa Cruz Biotech. Antibodies specific for β-actin (cat # 4967), IκBα (# 9247); histone 3 (# 9715); caspase 1 (# 3866); and IL-1β (# 2022) were purchased from Cell Signaling Technology (Danvers, MA). Anti-FLAG antibodies (# TA0011) were purchased from OriGene. FLAG-tagged proteins were immunoprecipitated using an immunoprecipitation kit (cat # A8592) purchased from Sigma-Aldrich (St Louis, MO). The tagged protein was eluted from the immunoprecipitation beads using the FLAG peptide as suggested by the supplier. Horseradish peroxidase (HRP) conjugated secondary anti-mouse (NXA-931) and anti-rabbit (NA-934) antibodies were from Amersham Biosciences.

### Reverse Transcriptase Reaction, Real-time PCR

Total RNA was isolated from THP-1 cells with Trizol reagent (Invitrogen, Carlsbad, CA, USA). cDNA synthesis, RT-PCR and quantitative real-time TaqMan PCR were performed as described previously [39]. The following primers were used for RT-PCR: hIL-1β (forward: 5′- CTCG CCAGTGAAATGATG GCT -3′; backward: 5′-GTCGGAGATTCGTAGCTGGAT- 3′); hAIM2 (Forward: 5′-ATGTG AAGCCGTCCAGA-3′; backward: 5′-CATCATTTCTGA TGGCTGCA-3′); IFI16 (forward: 5′-CCAAGACTGAAGACTG AA-3′; backward: 5′-ATCGT CAATGACA TCCAG-3′); and Actin (forward: 5′-GCTCGTCGTCGACAACGGCTC-3′; backward: 5′-CATGATCTGGGTCA TCTTCTC-3′). The following TaqMan gene-specific assays were purchased from the Applied Biosystems (Foster City, CA) and used as suggested by the supplier: AIM2 (Hs 00175457_m1), IFI16 (Hs 00194261_m1), ASC (Hs 00203118_m1), Caspase-1 (Hs 01554467_m1), IL-1β (Hs01555410_m1), and the endogenous control β-actin (assay Id# Hs99999903_ml).

### Cell Fractionations

Cytosolic and nuclear fractions were essentially prepared as described previously (40). Briefly, cell pellet were incubated for 10 min on ice in cell fractionation buffer (20 mM Tris-HCl, pH 7.5; 0.65% NP-40, 150 mM NaCl) containing protease and phosphatase inhibitors. The cytosolic fractions were collected by centrifuging the resultant cell lysate at 4000 rpm for 5 min in a refrigerated micro-centrifuge. The reaming nuclear pellet was washed with the cell fractionation buffer; the nuclear pellet was lysed by suspending into the RIPA buffer followed by brief sonication [39].

### Statistical Analyses

The measurement values are presented as means±SEM. The statistical significance of differences in the measured mean frequencies between the two groups was calculated using the Student's two-tailed *t* test.

## Results

### Expression of the IFI16 and AIM2 Proteins is Cell Type Dependent

**Figure 1:**
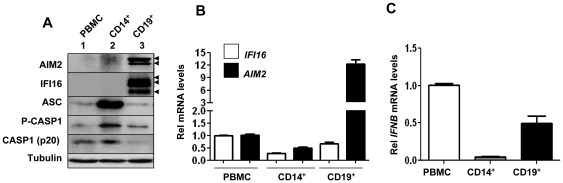
Expression of the IFI16 and AIM2 proteins is cell type dependent. (**A**) Total cell extracts containing equal amounts of proteins from peripheral blood mononuclear cells (PBMNC), purified monocytes (CD14^+^), or B lymphocytes (CD19^+^) were analyzed by immunoblotting using antibodies specific to the indicated proteins. The arrowheads indicate different forms of the AIM2 (53 and 49 kDa) and IFI16 (A, B, and C forms) proteins. (**B**) Total RNA was extracted from total PBMNCs, purified CD14^+^ or CD19^+^ cells. Steady state levels of the *IFI16* or *AIM2* mRNA were analyzed by quantitative TaqMan real-time PCR. The ratio of the test gene to *actin* mRNA was calculated in units (one unit being the ratio of the test gene to *actin* mRNA). Results are mean values of triplicate experiments and error bars represent standard deviation. (**C**) Total RNA as described in panel (**B**) was also analyzed by the quantitative TaqMan real-time PCR for the IFN-β mRNA. Again, the ratio of the test gene to *actin* mRNA was calculated in units. Results are mean values of triplicate experiments and error bars represent standard deviation.

An earlier study [41] reported that monocytic (CD14^+^) cells from peripheral blood stain positive for the expression of IFI16 protein in two color cytofluorography. Moreover, treatment of THP-1 cells, a monocytic cell line, with PMA was reported to increase steady-state levels of the AIM2, but not IFI16, mRNA [22]. Therefore, to begin to investigate the potential role of the IFN-inducible IFI16 protein in the regulation of the AIM2 inflammasome, we compared steady-state basal levels of the IFI16, AIM2, inflammasome proteins (ASC and procaspase-1), and IL-1β in peripheral blood-derived mononuclear cells (PBMCs), purified monocytes (CD14^+^), and purified B lymphocytes (CD19^+^). As shown in Fig. 1A, the basal steady-state levels of both IFI16 and AIM2 proteins were not detectable in the monocytes. However, the expression of both IFI16 and AIM2 proteins was readily detectable in the B lymphocytes. As expected [42], we detected all three isoforms (the A, B, and C) of the IFI16 protein in B lymphocytes. However, in contrast to our expectations [35], we detected two AIM2 protein bands in B lymphocytes, which migrated close to each other. Presently, it is not clear whether the slow-migrating protein band that was detected by the AIM2-specific antibody is a modified form of the AIM2 protein or an alternatively spliced isoform [15]. Moreover, the basal levels of the ASC and procaspase-1 were detectable in PBMCs and monocytes. Interestingly, we were able to detect the constitutive levels of activated caspase -1 (the p20 protein band) in extracts from PBMCs and monocytes, but not B lymphocytes.

Consistent with our above observations steady-state levels of the IFI16 and AIM2 mRNA were relatively lower in the monocytes as compared to B lymphocytes (Fig. 1B). Accordingly, levels of the IFN-β mRNA were lower in monocytes as compared to peripheral B cells (Fig. 1C). Together, these observations indicate that the basal levels of the IFI16 and AIM2 proteins depend on the immune cell-type and the peripheral blood monocytes express very low basal levels of IFI16 and AIM2 proteins.

### IFNs Differentially Induce the Expression of the AIM2 and Pro-IL-1β

**Figure 2:**
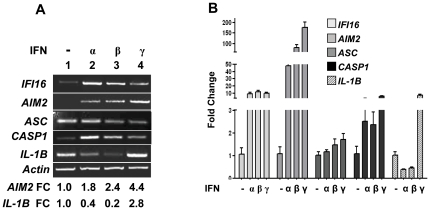
IFNs induce the expression of the IFI16, AIM2, and inflammasome genes. **(A**) Total RNA was extracted from THP-1 cells either left untreated (control) or treated with IFN-α (Universal IFN; 1000 u/ml), IFN-β (1000 u/ml), or IFN-γ (10 ng/ml) for 12 h. Relative steady-state levels of mRNA were analyzed by semi-quantitative RT-PCR using a pair of primer specific to the indicated genes. FC, indicates the fold change in the steady-state levels of the *AIM2* or *IL-1B* mRNA as compared to the control (lane 1). (**B**) Total mRNA in panel (**A**) was also analyzed for steady-state levels of mRNAs for the indicated genes by quantitative real-time PCR. The relative steady-state levels of the mRNA in control cells for each gene are indicated as 1. Results are mean values of triplicate experiments and error bars represent standard deviation.

The above observations that the basal levels of IFI16 and AIM2 proteins are not detectable in the peripheral blood monocytes prompted us to investigate whether IFN-treatment could up-regulate their expression. For this purpose, we chose to use THP-1 cells for several reasons: (i) these cells resemble the primary monocytes in that they constitutively express detectable levels of ASC adaptor protein and procaspase-1 [27]; (ii) cells are well-characterized with respect to inflammasome activation and its regulation [27], [37]; (iii) the expression of IFI16 protein can be induced by treatment of PMA-treated THP-1 cells with either type I or II IFNs [43]; (iv) a decrease in the AIM2 expression in THP-1 cells impaired dsDNA-induced maturation of IL-1β, which indicated that the endogenous AIM2 protein is required for DNA recognition [22]. Although, IFN-β treatment of THP-1 cells increases levels of the AIM2 mRNA [22], it remains unknown whether the IFN-β (or IFN-γ) treatment of these cells can also induce the expression of mRNA encoding for the IFI16 and/or inflammasome proteins, such as ASC and procaspase-1. As shown in Fig. 2, consistent with our above observations, basal levels of the IFI16, AIM2, and procaspase-1 mRNA were either very low or undetectable in untreated THP-1 cells. However, the basal levels of mRNAs encoding for the adaptor protein ASC and IL-1β were detectable. Interestingly, treatment of THP-1 cells with type I IFN (IFN-α or β) or type II IFN (IFN-γ) increased the steady-state levels of mRNAs encoded by the *IFI16*, *AIM2* and *P-CASP1* genes to different extents (Fig. 1A and 1B). The IFN-α treatment appreciably increased the steady-state levels of the IFI16 and CASP1 mRNA than the IFN-β or IFN-γ. In contrast to our expectations, the treatment of cells with IFN-γ increased levels of the AIM2 and pro-IL-1β mRNA measurably more than the type I IFNs. Importantly, the treatment of cells with type I IFNs (IFN-α or β) significantly decreased the steady-state levels of mRNA encoding for the pro-IL-1β (Fig. 1A and 1B). Together, these observations demonstrated that treatment of THP-1 cells with the type I or type II IFN increases steady-state levels of the IFI16, AIM2, CASP-1 mRNA whereas the treatment differentially regulates the levels of pro-IL-1β mRNA.

### IFNs and PMA Differentially Regulate the Expression of the IFI16, AIM2, and Inflammasome Proteins and Their Sub-cellular Localization

**Figure 3:**
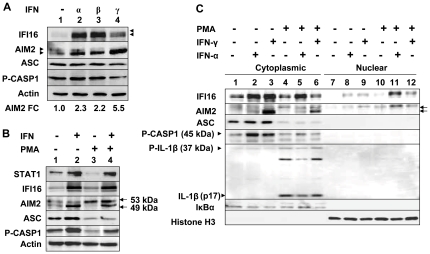
IFNs and PMA differentially regulate the expression of the IFI16, AIM2, and inflammasome proteins and their sub-cellular localization. (**A**) Total cell extracts were prepared from THP-1 cells either left untreated (control) or treated with IFN-α (Universal IFN; 1000 u/ml), IFN-β (1000 u/ml), or IFN-γ (10 ng/ml) for 12 h. Extracts containing approximately equal amounts of proteins were analyzed by immunoblotting using antibodies specific to the indicated proteins. The arrowheads indicate different isoforms of IFI16 protein. FC, indicates the fold change in the levels of the AIM2 protein with respect to the control (lane 1). (**B**) THP-1 cells were either left untreated (lane 1) or treated with IFN-α (1000 u/ml for 16 h) alone (lane 2), treated with PMA (1 µM for 3 h; lane 3) alone or both PMA and IFN-α (lane 4). After the treatment, total cell extracts containing approximately equal amounts of proteins were analyzed by immunoblotting using antibodies specific to the indicated proteins. The arrowheads indicate two protein bands (53 and 49 kDa) of the AIM2 protein. (**C**) THP-1 cells were either left untreated (lanes 1 and 7) or treated with IFN-α (1000 u/ml for 16 h) alone (lanes 2 and 8), IFN-γ (10 ng/ml for 16 h) alone (lanes 3 and 9), PMA (1 µM) alone (lanes 4 and 10), both PMA and IFN-α (lanes 5 and 11), or PMA and IFN-γ (lanes 6 and 12) . After the treatment, cells were fractionation into the cytoplasmic and nuclear fractions. The fractions containing approximately equal amounts of proteins were analyzed by immunoblotting using antibodies specific to the indicated proteins. Detection of the IκBα protein only in the cytoplasmic fraction and histone H3 in the nuclear fraction served as the quality control for the purity of cell fractionations.

The expression levels of IFI16 protein are also regulated by post-transcriptional mechanisms [42]. Therefore, we also analyzed basal and IFN-induced levels of the IFI16 and other proteins. As shown in Fig. 3A, consistent with our above observations, type I IFN treatment of cells induced IFI16 protein levels more than the IFN-γ treatment. Similarly, the IFN-γ treatment of cells induced levels of the AIM2 protein more than the type I IFNs. Interestingly, as noted above, we also detected two AIM2 protein bands in THP-1 cells. Again, consistent with our above observations, levels of the ASC and proscaspase-1 proteins were moderately (∼2-fold) increased by treatment of cells by type I IFNs. However, the treatment of cells with IFN-γ reduced the levels of both of these proteins by ∼50%. Moreover, as noted earlier [27], the expression of pro-IL-1β protein was not detectable in untreated or IFN-treated THP-1 cells (data not shown).

Treatment of THP-1 cells with phorbol 12-myristate 13-acetate (PMA) is reported to induce the expression of the *AIM2*, but not *IFI16*, gene [22]. Given that type I IFN (IFN-β) treatment of human diploid fibroblasts (HDFs) induces the expression of both AIM2 and IFI16 proteins [35], we treated THP-1 cells with PMA, IFN-α, or both and analyzed the expression levels of the IFI16 and AIM2 proteins. As shown in Fig. 3B, consistent with the previous report [43], treatment of cells with PMA alone did not induce the expression of IFI16 protein. However, the treatment increased levels of the AIM2 protein, which migrated slower than the form that was induced by the type I IFN (compare lane 3 with 2). Notably, both forms of the AIM2 protein were detected in cells that were treated with PMA and type I IFN (compare lane 4 with lanes 2 and 3). Interestingly, the treatment with PMA significantly decreased the basal levels of STAT1 transcription factor, adaptor protein ASC, and proscaspase-1. Moreover, treatment of cells with type I IFN along with PMA restored the expression of STAT1 and procaspase-1, but not the ASC protein (compare lane 4 with 3).

Sub-cellular localization of both IFI16 and AIM2 proteins appears to depend on the cell-type [15], [42]. Given that the IFI16 protein heterodimerizes with the AIM2 protein [17], [35], we investigated the sub-cellular localization of basal and induced levels of the IFI16, AIM2, and inflammasome proteins in THP-1 cells using cell fractionation approach. As shown in Fig. 3C, basal low levels of the IFI16, AIM2, ASC, P-CASP1, and pro-IL-1β proteins were detectable in the cytoplasmic fraction. Treatment of cells with IFN-α or γ, which induced levels of both IFI16 and AIM2 proteins, revealed that the bulk of the proteins were detected in the cytoplasmic fractions and only a small fraction was detectable in the nuclear fraction. Interestingly, it is the slow-migrating form of the AIM2 protein, which was detected in the nuclear fraction. Moreover, consistent with the previous reports [27], treatment of cells with PMA increased basal levels of pro-IL-1β and IL-1β proteins. Notably, the IFN-β treatment of PMA-differentiated THP-1 cells reduced the levels of the cleaved form of IL-1β (compare lane 5 with 4 or 6). Together, these observations revealed that IFNs and PMA differentially regulate the levels of IFI16, AIM2, and inflammasome proteins. Additionally, these observations indicated that the IFN-induced levels of both IFI16 and AIM2 protein are detected primarily in the cytoplasm.

### IFI16 Protein Binds to AIM2 Protein in the Cytoplasmic Fraction and Increased Levels of the IFI16 Protein Inhibit the AIM2-ASC Inflammasome-mediated Activation of Casapase-1

**Figure 4:**
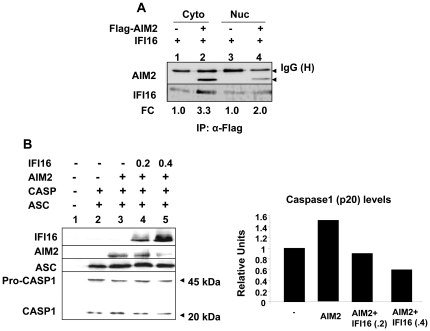
IFI16 protein binds to AIM2 protein in the cytoplasmic fraction and increased levels of the IFI16 protein inhibits the AIM2-ASC inflammasome-mediated activation of casapase-1. (**A**) Cultures of human embryonic kidney cells (HEK-293) at ∼80% confluency were either transfected with pCMV-AIM2 plasmid (2 µg) allowing the expression of the FLAG-tagged human AIM2 protein or along with the pCMV-IFI16 plasmid (1 µg) allowing the expression of IFI16 protein. 40 h after the transfections, cells were fractionated into cytoplasmic and nuclear fractions and fractions containing approximately equal amounts of proteins were subjected to immunoprecipitation using specific antibodies to the FLAG-tag. The immunoprecipitates were analyzed by immunoblotting using antibodies to the FLAG-tag (the upper panel) or the IFI16 protein (the lower panel). The fold change (FC) in the IFI16 protein levels in immunoprecipitates is also indicated. (**B**) Left panel: HEK-293 cells were either left untransfected (lane 1) or transfected with plasmid DNAs encoding for the ASC (DNA 50 ng; lanes 2–5), CASP-1 (DNA 100 ng; lanes 2–5), AIM2 (200 ng; lanes 3–5), or IFI16 (200 or 400 ng; lanes 4 and 5 respectively) proteins. Total amount of plasmid DNA in the transfections was adjusted to 2 µg using the pCDNA3 plasmid DNA. About 40 h after the transfections, cell lysates were analyzed by immunoblotting using antibodies specific to the indicated proteins. Right panel: Relative caspase-1 (p20) protein levels shown in the left panel (lanes 1–4).

The IFI16 protein heterodimerizes with the AIM2 protein [17], [35]. Therefore, detection of both IFI16 and AIM2 proteins in the cytoplasmic fraction as well as in the nuclear fraction of THP-1 cells prompted us to test their interactions in the two sub-cellular compartments. As shown in Fig. 4A, we were able to immunoprecipitate IFI16 protein that was bound to FLAG-tagged AIM2 protein in both cytoplasmic and nuclear fractions in transfected HEK-293T cells. Interestingly, consistent with our above observations (Fig. 3), more AIM2 protein bound to the IFI16 protein in the cytoplasmic fraction than the nuclear fraction. These observations prompted us to test whether increased expression of IFI16 protein could inhibit the AIM2-ASC-mediated activation of caspase-1. As shown in Fig. 4B, consistent with previous reports [27], [44], increased expression of adaptor protein ASC in transfected HEK-293T cells resulted in the generation of the p20 form of the caspase-1, thus, indicating its activation [27]. Moreover, co-expression of the FLAG-tagged AIM2 protein increased the cleavage of procaspase-1 (compare lane 3 with 2), which decreased the levels of procaspase-1 ∼50%. Importantly, increased expression of the IFI16 protein in HEK-293T cells reduced the activation of caspase-1 as determined by a reduction in the levels of the p20 form of the caspase-1 (compare lane 4 or 5 with lane 3). Together, these observations indicated that the IFI16 protein binds to AIM2 protein in the cytoplasmic fraction and increased expression of IFI16 protein in transfected cells can inhibit the AIM2-ASC-mediated activation of caspase-1.

### Knockdown of IFI16 Expression Increases the Constitutive Levels of the Activated Casapase-1

**Figure 5:**
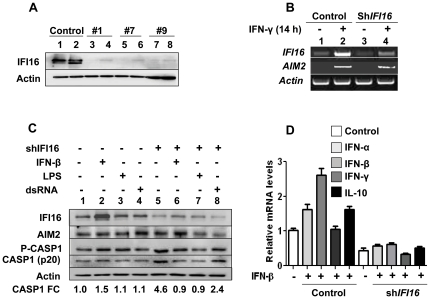
Knockdown of IFI16 expression increases the constitutive levels of the activated casapase-1. (**A**) Puromycin-resistant THP-1 cells either infected with control lentivirus (lanes 1 an2) or the virus expressing the shIFI16 RNA (lanes 3–8; cell populations from three different wells) were either left untreated (lanes 1, 3, 5, 7) or treated with IFN-α for 14 h. After treatment, total cell extracts containing increased amounts of proteins (∼100 µg/lane) were analyzed for the constitutive and induced levels of IFI16 and actin proteins. A long exposure was taken to detect the signal in all lanes. (**B**) Control THP-1 cells (lanes 1 and 2) or cell population from well # 9 (lanes 3 and 4) as shown in the panel (a) were either left untreated (lanes 1 and 3) or treated with IFN-α for 14 h (lanes 2 and 4). After the treatment, total RNA was analyzed for the steady-state levels of mRNA for the indicated genes. (**C**) Control THP-1 cells (lanes 1–4) or cells infected with virus expressing the shIFI16 mRNA (lanes 5–8; population # 9) were either left untreated (lanes 1 and 5) or treated with IFN-β (1,000 u/ml; lanes 2 and 6), LPS (100 ng/ml; lanes 3 and 7), or dsRNA (10 µg/ml; lanes 4 and 8) for 14 h. After the treatment, total cell lysates containing equal amounts of protein (∼100 µg/lane) were analyzed by immunoblotting using specific antibodies to the indicated proteins. FC, indicates the fold change in the levels of the activated caspase-1 (the p20 band) with respect to the control (lane 1). (**D**) Control THP-1 cells or cells infected with virus expressing the shIFI16 mRNA (population # 9) were either left untreated (white columns) or treated with IFN-β (1,000 u/ml; columns 2–5 and 7–10) for 14 h. After the treatment, total RNA levels were analyzed for the indicated genes by the quantitative TaqMan real-time PCR. The ratio of the test gene to *actin* mRNA was calculated in units (one unit being the ratio of the test gene to *actin* mRNA). Results are mean values of triplicate experiments and error bars represent standard deviation.

The above observations that increased levels of IFI16 protein in transfected cells inhibited the activation of caspase-1 by the AIM2-ASC inflammasome encouraged us to investigate whether the knockdown of the IFI16 expression in THP-1 cells could regulate the expression and/or functions of the AIM2 (or other) inflammasome. Therefore, we knockdown the expression of the IFI16 protein in THP-1 cells and isolated three independent cell populations (Fig. 5A). Moreover, as shown in Fig. 5B, the knockdown of IFI16 protein reduced basal (compare lane 3 with 1) and the IFN-induced (compare lane 4 with 2) levels of IFI16 mRNA. Importantly, the IFN-induced levels of the AIM2 mRNA did not decrease in THP-1 cells (Fig. 5B, compare lane 4 with 2). These observations allowed us to compare basal and induced (induced by IFN-β or LPS) levels of AIM2 and other inflammasome proteins between control THP-1 cells (infected with control virus) and cells in which we had constitutively decreased the basal levels of the IFI16 protein. As shown in Fig. 5C, consistent with our above observations (Fig. 5A) the knockdown of the IFI16 expression in THP-1 cells decreased basal and induced (induced by IFN-β, LPS, or double stranded RNA) levels of the IFI16 protein. Interestingly, the knockdown moderately (∼ 2-fold) increased the basal levels of AIM2 and P-CASP1 proteins (compare lane 5 with 1). Importantly, the knockdown increased the basal levels of the activated caspase-1 as determined by increases in the p20 protein band [27]. Together, these observations support the idea that the expression of IFI16 protein in THP-1 cells decreases the basal levels of the AIM2 and P-CASP-1, and the activation of CASP-1.

Induction of IFN-β expression in THP-1 cells by poly(dA:dT) treatment depends on the IFI16 expression [25]. Given that the IFN-β treatment of the murine BMDMs induced IL-10 expression in a STAT1-dependent manner and the increased production of IL-10 through activation of STAT3 transcription factor reduced the abundance of the pro-IL-1β [37], we explored whether the knockdown of IFI16 expression in THP-1 cells could negatively regulate the basal and IFN-β-mediated induction of IFNs and IL-10 expression. As shown in Fig. 5D, the knockdown of IFI16 expression in THP-1 cells decreased basal and IFN-induced levels of mRNAs encoding for the IFN-α, IFN-β, and IFN-γ. These observations are consistent with the previous report [25]. Notably, the knockdown of IFI16 expression also reduced the basal and the induced levels of the IL-10 mRNA. These observations revealed that the expression of IFI16 protein in THP-1 cells contributes to the expression of these cytokines.

### Knockdown of IFI16 Expression Increases the Activation of Caspase-1 by Inflammasomes

**Figure 6:**
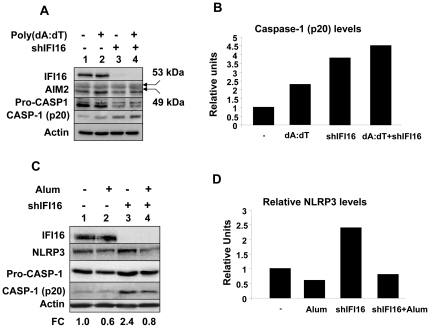
Knockdown of IFI16 expression increases casapase-1 activation by the AIM2 inflammasome. (**A**) Control THP-1 cells (lanes 1 and 2) or cells in which we knocked down the expression of IFI16 protein (lanes 3 and 4) were incubated with PMA (20 nM) for 14 h and then treated with LPS (1 µg/ml) for 3 h. Cells were then nucleofected without DNA (lanes 1 and 3) or with 2 µg of poly(dA:dT). After nucleofections, cells were incubated for 1.5 h and total cell lysates containing equal amounts of proteins were analyzed by immunoblotting using antibodies specific to the indicated proteins. The arrowheads indicate the bands corresponding to the indicated proteins. FC, indicates the fold change in the levels of the activated caspase-1 (the p20 protein band) with respect to the control lane 1. (**B**) Relative levels of caspase-1 (p20) protein shown in the panel A (lanes 1-4). (**C**) Control THP-1 cells (lanes 1 and 2) or cells in which we knocked down the expression of IFI16 protein (lanes 3 and 4) were incubated with PMA (100 nM) for 3 h and then treated with LPS (1 µg/ml) for 3 h. Cells were then either left untreated (lanes 1 and 3) or treated with alum (300 µg/ml) for 150 min. Total cell lysates containing equal amounts of proteins were analyzed by immunoblotting using antibodies specific to the indicated proteins. FC, indicates the fold change in the levels of the pro-caspase-1 with respect to the control lane 1. (**D**) Relative levels of NALP3 protein shown in the panel C (lanes 1–4).

Our above observations that the knockdown of IFI16 expression in THP-1 cells increases the constitutive levels of activated caspase-1 (the p20 form) encouraged us to test whether the reduced levels of the IFI16 protein in cells potentiate the activation of the AIM2 or NLRP3 inflammasome. Therefore, we used an optimized protocol [27] to study the activation of the AIM2 inflammasome by dsDNA poly(dA:dT). As shown in Fig. 6A, nucleofection of control or shIFI16 THP-1 cells (cells were treated with PMA overnight and then primed with LPS for 3 h) with the synthetic dsDNA increased the levels of activated caspase-1 (the p20) (compare lane 2 with 1 or lane 4 with 3). Consistent with our above observations, the constitutive levels of the caspase-1 (p20) were higher in the shIFI16 cells than the control cells (compare lane 3 with 1). Correspondingly, we detected lower levels of the procaspase-1 in THP-1 cells after the knockdown of IFI16 expression. Interestingly, the knockdown of IFI16 expression increased steady-state levels of the NLRP3 protein and the activated caspase-1 (compare lane 3 with 1). Moreover, treatment of the primed THP-1 cells with alum (an activator of the NLRP3), which decreased levels of the pro-caspase-1 (compare lane 4 with 2), also decreased levels of the p20 (compare lane 4 with 3). Together, these observations demonstrated that reduced levels of IFI16 protein in PMA-induced differentiated THP-1 cells potentiate dsDNA-mediated activation of the AIM2 inflammasome and alum-mediated activation of NLRP3 inflammasome.

## Discussion

IFN-β is being used in the clinic to treat several inflammation-associated diseases, including MS [6]–[8]. However, the molecular mechanisms by which the IFN-β exerts its anti-inflammatory effects remain largely unknown. Interestingly, inhibition of the production of proinflammatory cytokine IL-1β by type I IFN in immune cells provides a possible molecular basis for the anti-inflammatory action of the IFN-β in MS patients [37]. However, the IFN-inducible protein(s) that mediates the anti-inflammatory actions remain to be identified.

**Figure 7:**
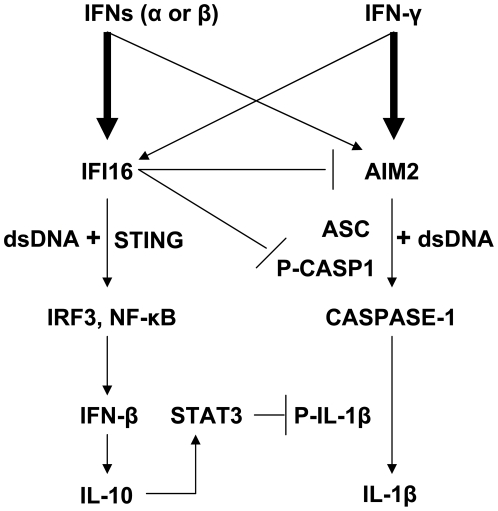
Type I and type II IFNs differentially regulate the expression of the IFI16 and AIM2 proteins. The expression of IFI16 protein suppresses activation of caspase-1 by the AIM2 inflammasome.

Given that: (i) the expression of both AIM2 and IFI16 proteins is induced by type I IFNs in certain cell types [15], [42]; (ii) upon sensing cytosolic dsDNA, only the AIM2, but not the IFI16, protein can form an inflammasome to activate caspase-1 [15], [25]; and (iii) both AIM2 and IFI16 proteins can form heterodimers [17], [35], we explored whether the IFI16 protein could negatively regulate activation of caspase-1 by the AIM2-ASC (or other) inflammasome. Our observations using the peripheral blood-derived monocytic cells (CD14^+^) and well-characterized THP-1 monocytic cell line revealed that (i) the expression of IFI16 and AIM2 proteins is cell type-dependent (Fig. 1); (ii) type I and II IFNs induce the expression of the IFI16, AIM2, and inflammasome proteins and corresponding mRNAs (Figs. 2 and 3); (iii) IFI16 protein binds to AIM2 in the cytoplasm and the increased levels of the IFI16 protein in transfected cells inhibit the AIM2-ASC inflammasome-mediated activation of casapase-1 (Fig. 4); (iv) the constitutive knockdown of the IFI16 expression in THP-1 cells increases the basal levels of the activated casapase-1 (Fig. 5); and (v) the knockdown of IFI16 expression increased activation of caspase-1 by the AIM2 and NLRP3 inflammasomes (Fig. 6). Together, these observations demonstrate that the IFN-inducible IFI16 protein suppresses activation of caspase-1 by the AIM2 and NLRP3 inflammasomes (Fig. 7).

Inflammasomes have been extensively characterized in monocytes, macrophages, and melanoma cells [27], [29], [45]. Moreover, a recent study has reported that the IFI16 protein can sense the KSHV DNA in the nucleus to activate caspase-1 and inflammasome activity in endothelial cells [32]. Furthermore, we recently noted that in senescent HDFs increased levels of the AIM2 protein are associated with the production of IL-1β [35]. Notably, the production of IL-1β was associated with reduced levels of the IFI16 protein. Accordingly, the knockdown of IFI16 expression in endothelial cells was associated with increased activation of caspase-1 by vaccinia virus [32]. Given that the knockdown of IFI16 protein expression in THP-1 cells increased basal and poly(dA:dT)-induced activation of caspase-1 (Figs. 5 and 6A), these observations are consistent with the recent report [32] and indicate a role for the IFI16 protein as a negative regulator of caspase-1 activation in a cells. Based on our observations, it is conceivable that the IFI16 protein exerts its anti-inflammatory functions by: (i) sequestering AIM2 protein through PYD (Fig. 4), thus, inhibiting the recruitment of ASC protein and the activation of the AIM2 inflammasome; and (ii) up-regulating the expression of IL-10 and IFN-β (Fig. 5D), anti-inflammatory cytokines. Because the expression of both IFI16 and AIM2 proteins is not limited to monocytes and macrophages (Fig. 1), it is likely that the ratio between IFI16 and AIM2 protein levels in the cytoplasm of a given cell type contributes to the decision between the production of type I IFNs and IL-1β (and possibly cell death, ref. 15) after sensing cytosolic dsDNA (Fig. 7).

Constitutive basal levels of mRNAs that encode for the IFI16, AIM2, and procaspase-1 were relatively low in THP-1 cells (Fig. 2). In contrast, the levels of mRNAs encoding for the pro-IL-1β were detectable. Interestingly, the basal levels of procaspase-1 protein were detectable in THP-1 cells whereas levels of pro-IL-1β were not detectable (Fig. 3). These observations suggest a post-transcriptional regulation of the levels of procaspase-1 and pro-IL-1β proteins in THP-1 cells.

The knockdown of p202 expression in BMDMs increased activation of caspase-1 by the AIM2 inflammasome [46]. Moreover, the levels of the p202 protein in BMDMs from non lupus-prone (B6) and a lupus-prone strain of mice (the NZB) were inversely correlated with the activation of caspase-1 by the AIM2 inflammasome [46]. Consistent with these observations, increased levels of the p202 protein in splenic and bone marrow-derived cells in lupus prone strains of mice were inversely correlated with the levels of the Aim2 protein and the production of pathogenic auto-antibodies [39], which are associated with the development of lupus-like disease [11]. Given that PBMCs from lupus patients express increased levels of the *IFI16* mRNA as compared to healthy individuals [47], our observations that the IFI16 protein suppresses the activation of caspase-1 by the AIM2-ASC inflammasome support the idea that the increased levels of the IFI16 protein in immune cells contribute to the development of autoimmunity. Further work is in progress to test this exciting possibility.

Previous studies using cells of the hematopoietic origin had indicated that the IFI16 protein is primarily localized in the nuclear compartment [48]. Moreover, a recent study has noted that the IFI16 protein is detected in the nucleus of endothelial cells [32]. However, in a human prostate epithelial cancer cell line (PC-3), the endogenous IFI16 protein was primarily detected in the cytoplasm [34]. Moreover, using a quantitative cell fractionation approach, we recently noted that the bulk of the IFI16 protein was primarily detected in the cytoplasmic fraction of normal HDFs and only a small fraction (∼25%) was detectable in the nuclear fraction [35]. Similarly, in THP-1 cells, the bulk of the IFI16 protein was detected primarily in the cytoplasmic fraction and only a small fraction (less than 10%) was detectable in the nuclear fraction (Fig. 3). Given that the IFI16 protein can heterodimerize with the AIM2 protein (and possibly other p200-family proteins) (Fig. 4; and ref. 17, 35), it is likely that interactions of the IFI16 protein with the AIM2 protein (and possibly with other p200-family proteins) contribute to its cytoplasmic localization. Notably, we have detected a small fraction (∼20%) of the AIM2 protein in the nuclear fraction of normal HDFs (35) and THP-1 cells (Fig. 3). Presently, it is not clear what regulates the nuclear localization of AIM2 protein in THP-1 cells. Given that IFI16 protein contains a classical nuclear localization signal and the protein heterodimerizes with AIM2 protein, it is likely that treatment of THP-1 cells with PMA and IFN-γ potentiates the nuclear localization of IFI16 protein and IFI16 protein “piggy backs” the AIM2 protein to the nucleus (Fig. 3C). Further work will be needed to address this interesting observation.

Type I IFN-treatment of the murine BMDMs induces IL-10 expression in a STAT1-dependent manner, thereby, reducing the abundance of the pro-IL-1β levels through the activation of STAT3 transcription factor [37]. Consistent with this observation, we noted that type I IFN-treatment of THP-1 cells reduced steady-state levels of pro-IL-1β mRNA (Fig. 2A), thus, raising the possibility that the IFN-inducible IFI16 protein increases the IL-10 expression. Interestingly, the knockdown of the IFI16 expression in THP-1 cells abrogated the type-I IFN-mediated increase in IL-10 mRNA levels (Fig. 5D). Therefore, we propose that the expression of IFI16 protein is required for the IFN-mediated induction of IL-10 expression and suppression of the pro-IL-1β expression. Moreover, treatment of THP-1 cells with PMA (which induced the levels of pro-IL-1β protein) and IFN-β decreased the levels of pro-IL-1β protein (Fig. 3). However, the knockdown of the IFI16 expression in THP-1 cells and treatment of cells with PMA did not result in increases in pro-IL-1β protein (Fig. 6). These observations indicate that the PMA-induced signaling pathways regulate the IFI16 mediated modulation of the pro-IL-1β expression in THP-1 cells. Therefore, further work is in progress to determine the molecular mechanisms by which the IFN-inducible IFI16 protein regulates the IL-10/STAT3/P-IL-1β pathway.

In summary, our observations demonstrate that type I and II IFNs up-regulate the expression of IFI16 and AIM2 proteins in THP-1 cells. However, the extent of induction by the two IFNs varies. Moreover, we found that type I IFN treatment of THP-1 cells decreases the levels of pro-IL-1β mRNA. Significantly, our observations demonstrate that the expression of the IFI16 protein in THP-1 cells suppresses activation of caspase-1 by the AIM2-ASC and NLRP3 inflammasomes. These observations will serve basis to investigate the role of IFI16 protein in the regulation of inflammation and associated diseases.
